# A Study of Pattern Prediction in the Monitoring Data of Earthen Ruins with the Internet of Things

**DOI:** 10.3390/s17051076

**Published:** 2017-05-11

**Authors:** Yun Xiao, Xin Wang, Faezeh Eshragh, Xuanhong Wang, Xiaojiang Chen, Dingyi Fang

**Affiliations:** 1 School of Information Science and Technology, Northwest University, Xi’an 710021, China; yxiao@nwu.edu.cn (Y.X.); xjchen@nwu.edu.cn (X.C.); dyfang@nwu.edu.cn (D.F.); 2 Department of Geomatics Engineering, University of Calgary, Calgary, AB T2N 1N4, Canada; feshrgh@ucalgary.ca; 3 Department of Communication, Xi’an University of Posts & Telecommunications, Xi’an 710121, China; wxh@xupt.edu.cn

**Keywords:** pattern prediction, multivariate sequential data, earthen ruin

## Abstract

An understanding of the changes of the rammed earth temperature of earthen ruins is important for protection of such ruins. To predict the rammed earth temperature pattern using the air temperature pattern of the monitoring data of earthen ruins, a pattern prediction method based on interesting pattern mining and correlation, called PPER, is proposed in this paper. PPER first finds the interesting patterns in the air temperature sequence and the rammed earth temperature sequence. To reduce the processing time, two pruning rules and a new data structure based on an R-tree are also proposed. Correlation rules between the air temperature patterns and the rammed earth temperature patterns are then mined. The correlation rules are merged into predictive rules for the rammed earth temperature pattern. Experiments were conducted to show the accuracy of the presented method and the power of the pruning rules. Moreover, the Ming Dynasty Great Wall dataset was used to examine the algorithm, and six predictive rules from the air temperature to rammed earth temperature based on the interesting patterns were obtained, with the average hit rate reaching 89.8%. The PPER and predictive rules will be useful for rammed earth temperature prediction in protection of earthen ruins.

## 1. Introduction

**Figure 1:**
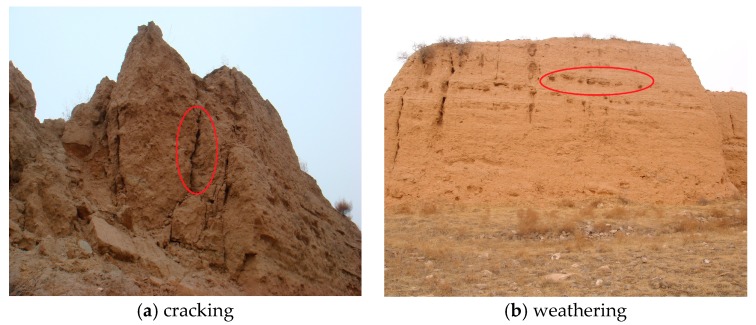
Cracking and weathering damage in Ming Dynasty Great Wall.

Earthen ruins are ancient ruins based on mud brick, rammed earth, or any type of construction using soil as the main building material and have great historical, cultural and scientific value [1]. Lacking the very architectural devices necessary for survival as originally designed (e.g., protective roofs and plastered surfaces), earthen ruins exist in a continual state of ‘unbecoming’ [2]. At the micro level, earth swelling and shrinkage coupled with decohesion of the earth-silt-sand agglomerate are the fundamental mechanisms responsible for soil destabilization, which will eventually lead to various scales of damage from the loss of surface and surface finishes. Most of these earthen ruins will either collapse over time from differential erosion, or eventually stabilize as unrecognizable lumps [2]. Figure 1 shows the cracking and weathering conditions (marked by circles) in the Ming Dynasty Great Wall in Shaanxi Province of China. Research shows that dramatic rammed earth temperature changes are one of the major reasons causing the destruction of earthen ruins [3,4]. Therefore an understanding of the changes of the rammed earth temperature of earthen ruins is important for protection of earthen ruins.

**Figure 2:**
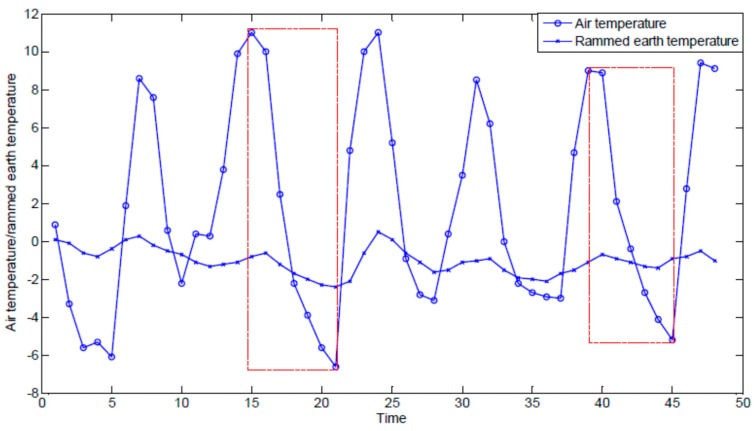
Air temperature and rammed earth temperature sequences.

The technology of the Internet of Things (IoT) has become an ideal solution for monitoring earthen ruins, because it is easily deployable and suitable for long-term and real-time data collection in remote areas. Most of the existing early studies have focused on collecting the monitoring data, but lack suitable data processing procedures [5,6,7]. Subsequent research has begun to focus on IoT monitoring data processing in the field of cultural heritage [8,9,10,11,12]. However, it is difficult for the earthen ruins conservation experts to make intelligent and scientific based decisions for protecting earthen ruins based on collected IoT data [13] due to the following challenges:

(1) It is difficult to describe the complex and interesting hidden relationship between internal earthen ruin parameters and external environmental parameters. For example, Figure 2 shows a plot of air temperature (line with ○) and rammed earth temperature of an earthen ruin (line with ×) sequences. As shown in the figure, in the area indicated by the left dashed box, both air temperature and rammed earth temperature values decrease. However, in the area indicated by the right dashed box, although the air temperature falls, the rammed earth temperature shows no significant change. In order to predict the rammed earth temperature trend based on the air temperature, we must know what kind of change in temperature is meaningful for earthen ruins conservation experts. However, not all the air temperature changes are interesting; for instance, earthen ruins conservation experts may only pay attention to air temperature changes that would cause damage in earthen ruins.
(2) With the increase in the number of IoT nodes, as well as the continuous monitoring, we will face dealing with huge amounts of time-series data. For example, the Ming Dynasty Great Wall ruins are more than 6000 km in length; if monitoring nodes were deployed every meter along the Ming Dynasty Great Wall, the number of nodes would be more than 6 million. Faced with large amounts of time-series data, we expect algorithms to provide prediction results efficiently.
(3) There is a hysteresis effect in rammed earth temperatures relative to air temperatures. Therefore, in the monitoring data of earthen ruins, a change in the rammed earth temperature has a certain delay relative to the change in air temperature. The delay parameter needs to be studied to be able to predict the exact rammed earth temperature trends with time.

To solve the aforementioned challenges, we propose a method called the Pattern Prediction on the monitoring data of Earthen Ruins (PPER) to acquire rammed earth temperatures of earthen ruins from IoT data. The proposed PPER can describe the interesting patterns of the earthen ruin variables by efficiently discovering the hidden relations between the time series of two correlated variables and pattern prediction. Specifically, the contributions of this paper are:

(1) Some terms for interesting patterns for earthen ruin monitoring are formalized. Terms, such as interesting pattern, direction, delay and variation of rule, are properly defined to precisely describe the patterns and changes in IoT data.
(2) The air temperature and rammed earth temperature are used as examples to show how the hidden relationships between the two earthen ruin monitoring data are discovered. Since dramatic temperature change is one major cause for the destruction of earthen ruins, we focus on the rising and falling of the temperature. PPER searches the whole dataset to find the rising and falling pattern and obtains the predictive rules to predict the rammed earth temperature.
(3) Two pruning rules are proposed to reduce the number of computations. At the same time, a new data structure version based on the R-tree [14] data structure is used to group similar patterns. Both techniques can significantly reduce the time complexity when processing huge amounts to IoT data.
(4) A set of experiments were conducted on the monitoring data of the Ming Dynasty Great Wall to demonstrate the effectiveness and efficiency of PPER. Interesting relationships between the air temperature and the rammed earth temperature were discovered to predict the rammed earth temperature pattern.

The rest of this paper is organized as follows: Section 2 briefly reviews the most related works to this study. In Section 3, the required definitions are presented. In Section 4, the proposed method is described in detail. Section 5 presents the experimental studies and the results. Finally, the concluding remarks and some ideas for further studies are discussed in Section 6.

## 2. Related Work

In this section, we present and discuss related studies on: (i) destructive, nondestructive and micro-destructive measurements for acquiring rammed earth temperatures of earthen ruins; (ii) IoT technologies in the cultural heritage domain, and (iii) time-series prediction methods.

### 2.1. Destructive, Nondestructive and Micro-Destructive Measurements for Earthern Site Monitoring

In order to acquire rammed earth temperatures of earthen ruins, there are a few options, which can be groups into destructive, nondestructive and micro-destructive measurements. With destructive measurement methods, such as using a dynamic nuclear polarization (DNP) series of temperature probes and digital thermometers, the rammed earth temperature can be accurately obtained, but the earthen ruin will be damaged to a certain degree [15]. Nondestructive measurement methods, such as infrared imaging systems, can be used to get the surface temperature of rammed earth [15,16]. Using this measurement method, only the rammed earth surface temperature can be obtained, and the internal temperature of the rammed earth is still unknown. The third type is micro-destructive measurement methods, such as measurements obtained using the IoT.

**Figure 3:**
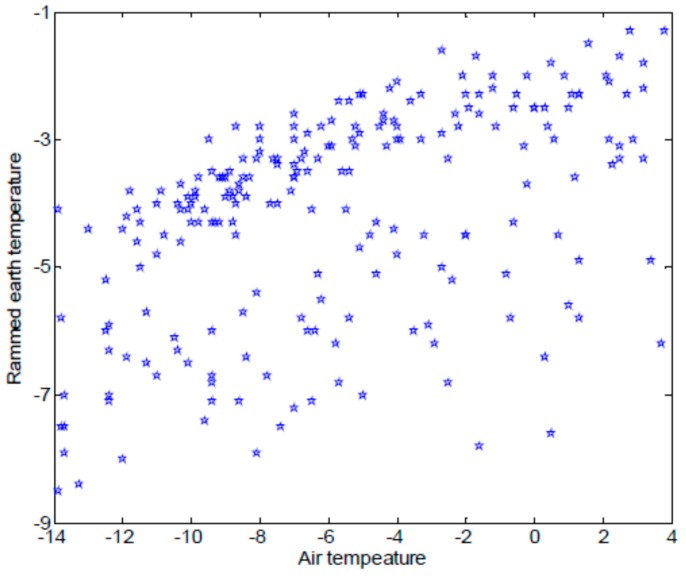
Relationship of the air temperature and the rammed earth temperature in Ming Dynasty Great Wall dataset.

Large amounts of monitoring data, such as the air temperature, can be obtained with IoT methods, such as the rammed earth temperature acquired by IoT nodes. A prediction model for IoT monitoring data based on a linear function between the air temperature and the rammed earth temperature was proposed to predict the rammed earth temperature of an earthen ruin in a completely closed environment [4]. Sufficient information on the rammed earth temperature with as little damage to the earthen ruin as possible was obtained. However, not all earthen ruins can be a completely closed environment, and the relation between the air temperature and the rammed earth temperature cannot always by satisfied with a linear function. For example, as shown in Figure 3, there is no simple linear relationship between the air temperature and the rammed earth temperature in the monitoring dataset of the Ming Dynasty Great Wall.

### 2.2. IoT Technology in Culture Heritage Domain

IoT technologies have made great progress in cultural heritage applications. These technologies have two phases, the data monitoring and the data processing phase.

In the data monitoring phase, for example, Abrardo and Rodriguez-Sanchez used the Internet of Things technology collect environmental information of heritage sites [5]. In addition, the Institute of Computing Technology Chinese Academy of Sciences has developed an intelligent environment monitoring system which collected environmental information such as temperature, humidity, light at the Palace Museum heritage site [6]. A new sensor network for indoor environmental monitoring has been developed [7].

In the data monitoring and data processing phase, there have been some interesting applications in the field of cultural heritage based on IoT technology. In [8], an IoT architecture was designed to support the design of a smart museum based on an innovative model of sensors and services. An intelligent IoT system, designed with the aim of improving user experience and knowledge diffusion within a cultural space, was presented in [9]. Typical IoT smart technologies represent an effective mean to support users’ understanding of cultural heritage [10]. An authoring platform, named FEDRO was presented automatically generate textual and users profiled artworks biographies, employed to feed a smart app for guiding visitors during the exhibition. An indoor location-aware architecture was designed and validated able to enhance the user experience in a museum [11]. In particular, the proposed system relies on a wearable device that combines image recognition and localization capabilities to automatically provide the users with cultural contents related to the observed artworks. A collaborative reputation system (CRS) was designed to establish the people reputation within cultural spaces [12].

### 2.3. Time-Series Prediction

Time-series prediction has many practical applications, such as weather forecasting and stock market prediction and therefore it has attracted a great deal of attention [17]. A large number of studies on time-series forecasting have utilized statistical models, such as autoregressive integrated moving average (ARIMA) [18] or exponential smoothing [19,20,21]. These models have been widely used for financial data analyses. However, the nonlinear and irregular nature of real-world time-series data has always been a problem. A newer approach to solving this problem uses machine learning techniques to predict the future results based on the knowledge learned from available data. For instance, artificial neural networks (ANN) have been widely used in time-series prediction [22,23,24]. However, disadvantages, such as over fitting and a time-consuming training phase, occur. Other techniques applied to solving this problem are support vector machines (SVM) [25] and the k-nearest-neighbor method [26].

Since many time-series variables are exposed to translation or dilatation in time, those approaches used traditionally for behavior forecasting will fail in providing useful hints about the future. A solution takes the behavior of the sequences into account rather than the exact values. For example, [27,28] proposed methodologies for predicting patterns in time series. Both of these studies suggested a new representation of data and then tried to find the most frequent patterns. However, these solutions had some problems. The data representation proposed in these solutions did not reduce the dimensionality of the data, especially for highly variant data. Therefore, a lot of data processing, like clustering, was necessary in [27], which can result in high time complexity.

Another problem was the inability to interpret the output rules and relationships. For instance, in [26] the relationship between patterns was defined using Allen’s interval relations [29], which did not provide enough information for calculating delay. Reference [28] had the same problem for extracting information, such as sensitivity and direction of the variations, since it used symbolic aggregation approximation (SAX) representation, which summarized the data sequences based on the average value of intervals and did not pay attention to direction of variations.

These approaches try to learn from previously seen or predicted data and then predict the future values. However, if they use predicted values as an input for the next prediction step, error accumulation problem is possible [17]. Moreover, in reality, it is highly probable that the values of a time series are influenced by variations of other groups of time series.

Studies about multivariate forecasting methods take this problem into account and aim at solving it using statistical or artificial approaches. Neural networks [30,31,32] are one of the most important techniques applied in this area. The proposed methods try to find the future value of a time series based on previously seen data. However, in many real world situations, we are more interested in the upcoming trend is of more interest, not the exact and specific values. To achieve this goal, some studies have been done on univariate time-series data, using a set of previously observed values, called a pattern, to predict a set of future value(s) [33,34,35,36]. These methodologies try to find the hidden trends in the data and predict the future using the discovered patterns.

Predicting multivariate time-series data using patterns has been studied with interval data knowledge discovery [27,28,32]. Hoppner’s work was aimed at solving a problem similar to the current study [27]. The author tried to find frequent patterns and use them for mining hidden rules in the data. However, their approach was different from our methodology in some aspects. They partitioned the sequences based on different data trends and coded the data using these intervals. However, this representation of the data may not reduce the complexity of the data, especially when the variations are very frequent. Moreover, clustering of similar intervals is a highly complex step, due to the large number of intervals and the sequential nature of the data.

Our proposed method solves these problems by considering only intervals of the data that are of higher importance to the user, instead of all partitions. Additionally, since Hoppner’s method used Allen’s interval relations [29], it did not take the pattern locations into account and could not, therefore, extract important information, such as delay and direction of the relationships, which are essential for decision making. We solve this problem using concepts such as pattern location and slope.

## 3. Definitions and Notations

In this section, all air and rammed earth monitoring data can be represented as sequential data.

**Definition 1** (Sequence)**.** *A sequence* $S=\{s_{1},\cdots ,s_{l}\}$ *is an ordered set of values, where* $s_{i}$ (1≤i≤l) *is a value of a sequence variable, and* l *is length of the sequence*.

In this regard, each value shows a data point (e.g., in time or space). For each data point, *i* indicates the index of the data point in the ordered set S. Time-series data are a special case of sequential data where the order is temporal. For example, S={4.6,4.3,4.1,4.4,7.3,8.5,6.6,5.3} is a daily rammed earth temperature sequence measured every three hours, with *l* as 8. $s_{1}$ is 4.6, which means the rammed earth temperature is 4.6 °C at 0:00, and so on.

**Definition 2** (Subsequence)**.** *Sequence* $S^{\prime }=\{s_{I},\cdots s_{I+k-1}\}$ *is called a subsequence of S if* $S^{\prime }\subseteq S$, *and* 1≤I<⋯<I+k−1≤l. *Here* k *is the length of the subsequence, and the subsequence* $S^{\prime }$ *is an ordered subset of* S *starting from start index* I.

For example, in sequence S, subsequence $S^{\prime }=\left\{s_{3},s_{4},s_{5},s_{6}\right\}=\left\{4.1,4.4,7.3,8.5\right\}$. I is 3, and k is 4.

**Definition 3** (Variance of sequence)**.** *Given a sequence* S, *the variance of* S *in term of the first element is defined as:*

(1) $$\delta \left(S\right)=\frac{1}{n}\overset{j=l-1}{\underset{j=1}{\sum }}{\left(s_{j+1}-s_{1}\right)}^{2}$$

Sequence variance measures how far sequence values are spread out. We are interested in the amount of changes from the first element of the sequence ($s_{1}$); for sequence S={4.6,4.3,4.1,4.4,7.3,8.5,6.6,5.3}, then $\delta \left(S\right)=\left({\left(4.3-4.6\right)}^{2}+{\left(4.1-4.6\right)}^{2}+{\left(4.4-4.6\right)}^{2}+{\left(7.3-4.6\right)}^{2}+{\left(8.5-4.6\right)}^{2}+{\left(6.6-4.6\right)}^{2}+{\left(5.3-4.6\right)}^{2}\right)/8\approx 3.4$. For $S^{\prime }=\left\{4.1,4.4,7.3,8.5\right\}$, $\delta \left(S^{\prime }\right)=\left({\left(4.4-4.1\right)}^{2}+{\left(7.3-4.1\right)}^{2}+{\left(8.5-4.1\right)}^{2}\right)/4\approx 7.4$.

**Definition 4** (Slope of sequence)**.** *Slope m of a sequence is defined as:*

(2) $$m\left(S\right)=\frac{s_{l}-s_{1}}{\left|s_{l}-s_{1}\right|}=\{\begin{array}{c} 1,\;\;\;\;\;\;s_{l}>s_{1}\\ 0,\;\;\;\;\;\;\;s_{l}=s_{1}\\ -1,\;\;\;\;s_{l}<s_{1} \end{array},$$

*The slope of a sequence describes the rising or falling direction of the sequence. If the slope is positive, the sequence is rising. If the slope is negative, it is falling. For sequence* ={4.6,4.3,4.1,4.4,7.3,8.5,6.6,5.3}, *since* 5.3>4.6, *then* m(S)=1. *For subsequence* $S^{\prime }=\left\{4.1,4.4,7.3,8.5\right\}$, *since* 8.5>4.1, *then*$\;m\left(S^{\prime }\right)=1$.

**Definition 5** (Multivariate sequence)**.** *Consider n number of sequence* $S_{1},S_{2},\cdots S_{n}$. *A multivariate sequence* Y *is a set of sequences denoted as* $Y=\left(\begin{array}{c} \begin{array}{c} S_{1}\\ S_{2} \end{array}\\ \begin{array}{c} \vdots \\ S_{n} \end{array} \end{array}\right)$. *For index i*, $Y_{i}=\left(\begin{array}{c} \begin{array}{c} S_{1i}\\ \vdots \end{array}\\ s_{ni} \end{array}\right)$ *where* $s_{ji}$ *indicates the ith data point of* $S_{j}$.

For example, $S_{1}$ is a sequence of air temperature and $S_{2}$ is a sequence of rammed earth temperature, then $Y=\left(\begin{array}{c} S_{1}\\ S_{2} \end{array}\right)=\left(\begin{array}{rrrrrrrr} 8.5, & 7.3, & 7.1, & 9, & 15.1, & 14.8, & 11.6, & 8.4\\ 4.6, & 4.3, & 4.1, & 4.4, & 7.3, & 8.5, & 6.6, & 5.3 \end{array}\right)$ as a multivariate sequence formed from the monitoring data. Multivariate sequential analysis is used to model and explain the interactions and co-movements among a group of sequence variables.

**Definition 6** (Interesting pattern)**.** *Given sequence S of length l, subsequence* $S^{\prime }$ *of* S *is interesting pattern* p *if its variance is greater than a threshold* $\delta_{min}$, *i.e.*, $\delta \left(S^{\prime }\right)\geq \delta_{min}$.

In this paper, we are interested in finding the rising and falling patterns in the observed air and rammed earth temperature data. We define the concept of interesting pattern based on the variance.

A group of interesting patterns discovered from a set of sequences is called a candidate pattern set. For example, given $\delta_{min}=5$, $p_{air\_3}$ is an interesting pattern discovered from the air temperature sequence of an earthen ruin, because $S_{air\_3}^{'}=\left\{10.2,\;\;8.8,\;\;5.6,\;\;3.4,\;\;1.2\right\}$ and $\delta \left(S_{air\_3}^{'}\right)\approx 30.1>5$. Given $\delta_{min}=1$, $p_{earth\_5}$ is an interesting pattern discovered from the rammed earth temperature sequence, because $S_{earth\_5}^{'}=\left\{3.1,\;\;2.4,\;\;2.1,\;\;1.5,\;\;0.1\right\}$ and $\delta \left(S_{earth\_5}^{'}\right)\approx 4.1>1$.

Given a set of variables, variables (e.g., air temperature) that can be used for predicting other variables are called conditional variables. The other variables that can be predicted using conditional variables are called decision variables (e.g., rammed earth temperature). In this paper, we study how to predict rammed earth temperature with air temperature, so an important concept is the correlation rule.

**Definition 7** (Correlation rule)**.** *Let* P *be a set of candidate pattern sets related to condition al variables in a dataset of n multivariate sequential data*, $P^{\prime }$ *be another set of candidate pattern sets related to decision variables*, *and* P *and* $P^{\prime }$ *are complement regarding the whole set of candidate pattern sets of the dataset. The correlation rule is defined as the form* $r=p\Rightarrow p^{\prime }$ *where* p∈P *and* $p^{\prime }\in P^{\prime }$.

For example, since we focus on prediction of rammed earth temperature with the air temperature of earthen ruins, the correlation rule will be like this $r=p_{air\_3}\Rightarrow p_{earth\_5}$. In this application, earthen ruins conservation experts are interested in not only the combination of the conditional variable patterns influence the decision variables, they are also interested in how the decision variables such as the rammed earth temperature corresponding to every single conditional variable. Therefore, we introduce the following concepts of direction, delay and variation of the rules to describe the possible relationships between single conditional variable and the decision variable. .

**Definition 8** (Direction of the rule)**.** *Given rule* $r=p\Rightarrow p^{\prime }$, *the direction of rule* r *is defined as:*

(3) $$D\left(r\right)=m\left(p\right)\times m\left(p^{\prime }\right)=\{\begin{array}{cc} 1, & m\left(p\right)=m\left(p^{\prime }\right)=1\;or\;m\left(p\right)=m\left(p^{\prime }\right)=-1\\ 0, & m\left(p\right)=0\;or\;\;m\left(p^{\prime }\right)=0\\ -1, & otherwise \end{array},$$

Here, a positive result means the corresponding variables in the patterns’ time intervals move together (positive correlation), while a negative value means opposite movements of the variables in the patterns’ time intervals (negative correlation). Otherwise, the direction of the rule is defined as zero.

**Definition 9** (Delay of the rule)**.** *Given rule* $r=p\Rightarrow p^{\prime }$, *let* p.I *means the start index value of pattern* p, *the delay of rule* r *is defined as:*

(4) $$\Delta \left(r\right)=p.I-p^{\prime }.I$$

*The delay explains how long it takes to see the effects of changes of one variable in the value of another variable. For* $r=p_{air\_3}\Rightarrow p_{earth\_5}$, *start index I of* $p_{air\_3}$ *is 296, start index I of* $p_{earth\_5}$ *is 295, then* Δ(r)=296−295=1. *If the monitoring frequency is every 3 hours, then* Δ(r)=1 *means the delay is 3 h*.

**Definition 10** (Variation of the rule)**.** *Given rule* $r=p\Rightarrow p^{\prime }$, *let* max(p) *and* min(p) *be the minimum and maximum values of the elements in pattern* p, *the variation of rule* r *is defined as a pair* (*V*(p),*V*($p^{\prime }))$, *where:*

(5) V(p)=(max(p)−min(p)) ×m(p),

(6) $$V(p^{\prime })=\left(\max\left(p^{\prime }\right)-\min\left(p^{\prime }\right)\right)\times m\left(p^{\prime }\right),$$

*The variation of the rule indicates the degree of changes in terms of one variable in the value of another variable. For example, given rule* $r=p_{air\_3}\Rightarrow p_{earth\_5}\;\;$, *because* $S_{air\_3}^{\prime }=\left\{10.2,\;\;8.8,\;\;5.6,\;\;3.4,\;\;1.2\right\}$ *and* $S_{earth\_5}^{\prime }=\left\{3.1,\;\;2.4,\;\;2.1,\;\;1.5,\;\;0.1\right\}$, $max\left(p_{air\_3}\right)=10.2$, $min\left(p_{air\_3}\right)=1.2$,$\;max\left(p_{earth\_5}\right)=3.1$ *and* $\min\left(p_{earth\_5}\right)=0.1$. $V(p_{air\_3})=-9$ *and* $V(p_{earth\_5})=-3$; *thus, the variation of the rule is (−9, −3)*.

## 4. The PPER Algorithm

In this section, we present the proposed PPER algorithm, which can be viewed as a three-stage process: (1) find interesting patterns; (2) generate predictive rules; and, (3) predict with these predictive rules.

The first stage of PPER is finding interesting patterns that summarize data behavior. In this study, we are specifically interested in variations of the data, so we are looking for rising and falling patterns. Since sequential data may contain repetitive patterns, then the algorithm is to identify similar patterns and group them together. For each group of similar patterns, a representative pattern will be defined. All representative patterns together form a filtered pattern set. The second stage of PPER is using conventional data mining algorithms to retrieve the correlation rules with the filtered pattern set. Then comes filtering and merging, where the correlation rules are then filtered and merged into predictive rules based on earthen ruins prediction requirements. The predictive rules are used to predict the rammed earth temperature of the earthen ruins in the last stage of PPER. The remainder of this section describes the major steps of the algorithm in detail.

### 4.1. Finding Intersting Patterns

The first stage of PPER algorithm is to find interesting patterns that summarize the data behavior. It includes two steps: (1) identification of the candidate pattern set; and, (2) grouping of similar patterns.

#### 4.1.1. Identifying the Candidate Pattern Set

For each variable in the dataset (e.g., air temperature and rammed earth temperature variables in the earthen ruin monitoring dataset), the algorithm explores all the sequences to find the rising and falling patterns. For each sequence, it starts with a sliding window with a prespecified size from the first data point of the sequence. It then calculates the variance of the subsequence based on Definition 3 and checks whether the variance is greater than a threshold ($\delta_{min}$). If the variance is less than $\delta_{min}$, then there is no interesting pattern in the sliding window and the sliding window is discarded.

Interesting patterns may overlap, for example, the end point of the previous interest pattern is the starting point of a later interesting pattern. Therefore, the partially overlapping window is slid across the sequence and continues with a new sliding window. If the sliding window meets the variance condition, the algorithm continues to extend the sliding window to find the pattern with the maximum possible length. The extension starts by adding the next data point of the sequence to the sliding window. The algorithm continues adding points while the variance keeps increasing and the slope of the last added points is the same as the starting slope. After the extension, the data in the sliding window and its origin information (the identifier of the corresponding sequence) are used to form a pattern. The algorithm then skips those data points and continues finding patterns in the rest of the sequence.

The algorithm is described below. In Algorithm 1, the number of variables of the dataset and the length of each interesting pattern is usually very small, so the time complexity impact caused by them can be ignored. Therefore, the time complexity of the algorithm is O(n), where n is the number of sequences. Lines 1 to 7 check whether the variance is greater than a threshold ($\delta_{min}$). If the variance is more than $\delta_{min}$, lines 8 to 18 show how to find the longest pattern, and lines 10 to 12 show the details of the extension.

**Algorithm 1.** FindCandPatterns Algorithm.**FindCandPatterns (Dataset, Window Size,**
$\delta_{min}$, **Overlapping Length)****Input:** Dataset, Window Size, $\delta_{min}$

**Output:** candidate pattern set CandSet  1: Initiate variables and parameters CandSet = ɸ, w = Window Size , o = Overlapping Length;  2: **For** each Set *i* related to variable *i* in the dataset  3:   **For** each *S* in Set *i*
 4:     start = 0;  5:     **While**(start < *S*.length)  6:       SW = $S_{start}^{w}$; // initiating sliding window  7:       **If** SW.variance > $\delta_{min}$
 8:         new = SW ∪ $\left\{s_{\mathrm{SW}.\mathrm{end}+1}\right\}$; // adding the next data point in S to SW  9:         **while**
$\delta_{new}>\;\delta_{SW}$ and slope(new) = = slope(SW)  10:          SW = SW ∪ $\left\{s_{\mathrm{SW}\cdot \mathrm{end}+1}\right\}$;  11:        **EndWhile**
 12:        P = {SW,SID };  13:        CandSet = CandSet ∪ P;  14:        start = start + P.length;  15:       **Else**
 16:        start = start + o;  17:       **EndIf**
 18:     **EndWhile**
 19:   **EndFor**
 20: **EndFor**
 22: **Return** CandSet

#### 4.1.2. Grouping Similar Patterns

After discovering all the candidate patterns of different sequence groups in the dataset, we use these patterns to obtain the hidden relationships between the conditional variable (air temperature in this case) and decision variable (rammed earth temperature). However, for real earthen ruin monitoring datasets with a large number of long sequences, the number of candidate patterns may be very large. As a result, using this collection of patterns for prediction purposes (in the next stage) may not only be computationally expensive in terms of time and space requirements, but also can result in sparse and meaningless predictions. Moreover, the behavior of each sequence may be very similar in different intervals. Such similar behavior may result in the discovery of many similar patterns for one variable. Therefore, we tend to find and filter such similar patterns in the discovered pattern collection to reduce the size of the pattern set and avoid doing repetitive computations for similar patterns. In other words, for every group of similar patterns, we try to determine a representative pattern and use this representative in the next stages of the algorithm.

The first step in finding similar patterns is to generate a distance matrix between patterns. For each pair of patterns, an element of the matrix shows the distance between the two patterns. Therefore, if P is the set of candidate patterns, the distance matrix is a |P|*|P| matrix. A naïve algorithm can be used to calculate the distance between all pairs of patterns. However, the time complexity of generating the matrix is $O\left({\left|P\right|}^{2}\right)*O\left(f\left(dist\right)\right)$, where |P|, is the number of patterns identified and O(f(dist)) is the time complexity of the distance function (e.g., dynamic time warping (DTW) or Euclidian distance). It is obvious that the naïve algorithm is highly time consuming, regardless of the distance function, since applying distance measurements on sequential data requires exploring all the data points of the sequences. To tackle this problem we extend and utilize a new data structure based on an R-tree [14] and some pruning rules.

##### Data Structure

An R-tree is a tree-based data structure that is very popular for indexing spatial data [14]. Unlike Quad-tree, R-tree does not require non-spatial attribute to divide the space and can get better performance without fine tuning the tiling level [37]. The key idea of the data structure is that it represents a group of close data points determined using a minimum bounding rectangle (MBR) and then indexing the MBRs by applying a hierarchical structure. Since the original R-tree was designed for spatial data, it cannot be directly applied to the sequential data. In this study, we modified the R-tree concepts, so that we can use them for indexing subsequences.

**Figure 4:**
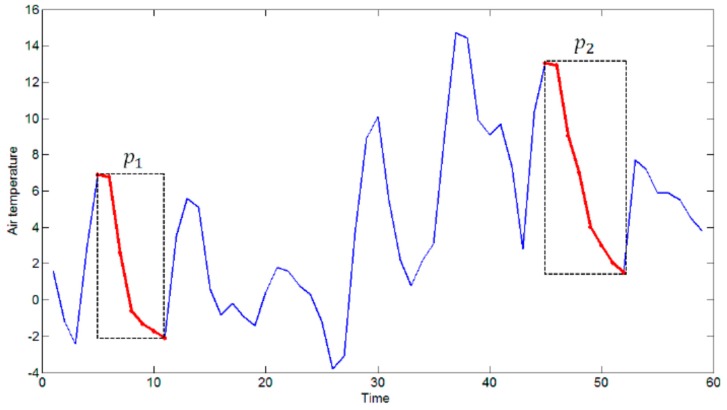
MBR of patterns.

Based on the first step of the algorithm, we have the start index I and length n of each pattern p in the candidate pattern set. We also have all the elements of the pattern subsequence. To be able to use the R-tree, the index of the sensor reading is mapped to the x coordinate and the value of the reading to the y coordinate. Therefore, for each pattern p in the candidate pattern set P, we can build an MBR where the corners are (I, min(p)) and (E,max(p)). E is the end index of the pattern p, and E=I+n−1. For example, the start index *I* of $p_{1}$ is 5, the length n of $p_{1}$ is 8; therefore, the end index E=12. Figure 4 shows the MBRs of patterns $p_{1}$ and $p_{2}\;$.

**Figure 5:**
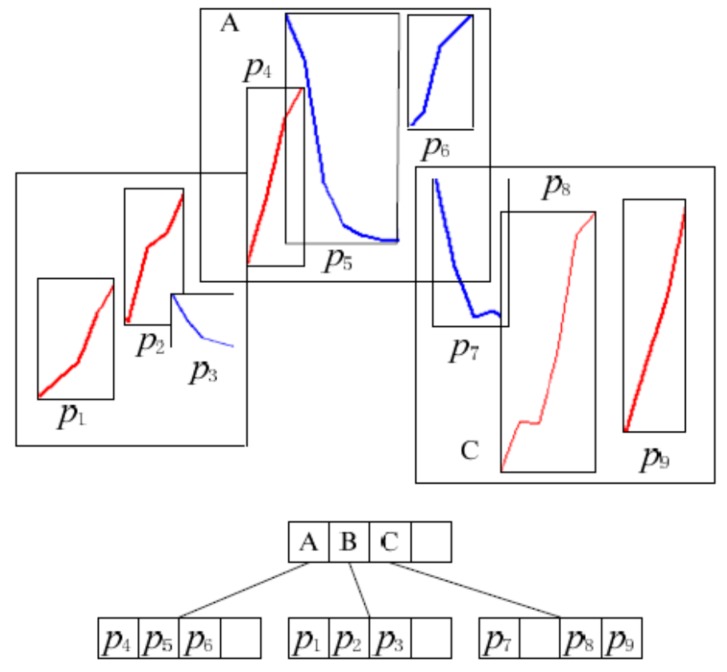
R-tree structure for indexing candidate patterns.

As a result, candidate pattern set P can be indexed using a modified R-tree, as shown in Figure 5, where each leaf is an MBR of a pattern and the intermediate entries of the R-tree index patterns with nearby MBRs.

This new data structure is used for reducing the time complexity by pruning the number of processed patterns.

**Definition 11** (Minimum distance between patterns)**.** Given two MBRs of two patterns A and B, the minimum distance between the two patterns is defined as:

(7) $$d\left(A,B\right)=\sqrt{d_{minX}{\left(A,B\right)}^{2}+d_{minY}{\left(A,B\right)}^{2}}$$

*where* $d_{minX}\left(A,B\right)$ *is defined as the minimum distance between projections of* A *and* B *in the x dimension and* $d_{minY}\left(A,B\right)$ *is the minimum distance between projections of* A *and* B *in the y dimension*.

**Figure 6:**
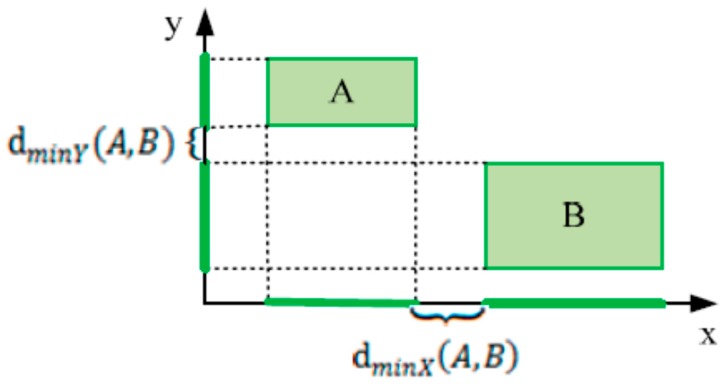
Minimum distance between projections of *A* and *B* in x and y dimensions.

Figure 6 illustrates an example of the minimum distance for x and y dimensions of two MBRs A and B.

##### Pruning-Based Calculation of Distance Matrix

In order to quickly and efficiently calculate the distance between pattern pairs, we apply the following two pruning rules in the algorithm of pruning-based calculation of distance matrix:

(1) *Pruning rule 1*. If two similar patterns $p_{1}$ and $p_{2}$ do not appear in the area$\;\left(I-W_{s},E+W_{s}\right)$ of the sequences, then the distance of the two patterns is infinity. $W_{s}$ is a user specified parameter. Pruning rule 1 is based on the fact that two similar patterns, far from each other, are considered to be two distinct patterns. As a result, there is no need to calculate their distance, and we can simply fill the corresponding matrix element by infinity.
(2) *Pruning rule 2*. If pattern $p_{1}$ is falling (negative slope) and pattern $p_{2}$ is rising (positive slope), then the distance of the two patterns is infinity. Pruning rule 2 uses for pruning the slope of the patterns. If one pattern is falling (negative slope) and the other one is rising (positive slope) they cannot be considered as similar and therefore we do not need to calculate their similarity. That is, we just simply fill the corresponding matrix element by infinity.

The process of pruning-based calculation of the distance matrix starts from the root node of the tree. For the set of roots’ children, it extracts all combinations of two are extracted. For each pair (e.g., $ch_{1},ch_{2}$), whether the pair elements are patterns or intermediate nodes is first checked. For intermediate nodes, with the first element of the pair ($ch_{1}$), and the algorithm of pruning-based calculation of distance matrix is used on the combinations of $ch_{1}$’s children. The same process is also done for the second element of the pair. We then decide whether we should compare $ch_{1}$’s children with $ch_{2}$’s children. To do so, the algorithm calculates the minimum distance between the MBRs of $ch_{1}$ and $ch_{2}$. If the distance is larger than the distance threshold, it means that the two groups of patterns indexed by the MBRs of $ch_{1}$ and $ch_{2}$ are either happening far from each other, or the element’s values are not close enough. Therefore, we can skip calculating the exact distance between these patterns and fill the corresponding elements of the distance matrix with infinity according to pruning rule 1. In the other case, where the minimum distance between $ch_{1}$ and $ch_{2}$ MBRs is less than the threshold, the algorithm continues with the combinations of $ch_{1}$ and $ch_{2}$’s children.

The process of pruning-based calculation of the distance matrix proceeds until it reaches the leaves of the tree, which means the elements of the input pairs are patterns. It applies two pruning rules to fill the corresponding element of the distance matrix with infinity. If neither of the pruning rules works, the algorithm calculates the distance between two patterns and fills the corresponding element of the distance matrix. Algorithm 2 details the process for calculating the distance matrix. Lines 2 to 4 show how to get the children list of the root. For each pair (e.g., $ch_{1},ch_{2}$), line 6 checks whether the pair elements are patterns or intermediate nodes. Lines 7 to 13 show how to fill the corresponding element of the distance matrix according to the two rules.

**Algorithm 2.** CalDistMatrix Algorithm.**CalDistMatrix (Pattern set P)****Input**: Pattern set P 
**Output**: distance matrix DistanceMatrix  1: Initiate DistanceMatrix with infinty;  2: Rtree patternTree = P.index();  3: root = patternTree.getRoot;  4: childrenList = root.getChildren();  5: **For** each (*ch*_1_ , *ch*_2_) in childrenList  6:   **If**
*ch*_1_,*ch*_2_ are not patterns  7:     getChildren(*ch*_1_);  8:     **do**
 9:     **If** the children *i* and *j* of *ch*_1_ are in same area and with same slope  10:      DistanceMatrix[*ch*_i_.id][ *ch*_j_.id] = dist(*ch*_i_,*ch*_j_);  11:    **EndIf**
 12:    **While** patterns are found;  13:    getChildren(*ch*_2_);  14:    **do**
 15:    **If** the children *k* and *l* of *ch*_2_ are in same area and with same slope  16:      DistanceMatrix[*ch*_k_.id][ *ch*_l_.id] = dist(*ch*_k_,*ch*_l_);  17:    **EndIf**
 18:    **While** patterns are found;  19:    **If** MBRdist(*ch*_1_,*ch*_2_)<${\;d}_{\mathrm{threshold}}$ and ch_1_ and ch_2_ are in same area and with same slope  20:      DistanceMatrix[ch_1_.id][ch_2_.id] = dist(ch_1_,ch_2_);  21:    **EndIf**
 22:   **EndIf**
 23: **EndFor**
 24: **Return** DistanceMatrix

##### Finding Similar Patterns

After calculating the distance matrix, the algorithm uses the matrix to decide which patterns are similar. For each pair of patterns $\left(p_{i},p_{j}\right)$, if their distance is less than distance threshold $\tilde{d}$, then are similar; and, the more representative one needs to be kept. We consider the one with highest number of similar patterns as the representative one (e.g.,$\;p_{i}$) because it covers a wider range of similar patterns and represents a larger population. Therefore, $p_{j}$ should be added to the set of similar patterns of $p_{i}$ and then removed from the result list. Continuing this process results in a reduced set of patterns, which can be used for the next step of the algorithm.

Algorithm 3 shows the algorithm to find similar patterns. Line 4 shows which patterns are similar if their distance is less than $\tilde{d}$. Lines 5 to 12 show how to get the longest pattern to represent other similar patterns.

**Algorithm 3.** FindSimilarPatterns Algorithm.**FindSimilarPatterns( Pattern set P,**$\;\tilde{d}$**)****Input**: Pattern set P, $\tilde{d}$ **Output**: filteredPatternList  1: filteredPatternList = P;  2: **For**
*i* = 0 to P.size  3:   **For**
*j* = 0 to P.size  4:     **If** DistanceMatrix(i,j) < $\tilde{d}$
 5:       **If** P(*i*).similarlist.size() > P(*j*).similarlist.size()  6:         P(*i*).similarlist.add(P(*j*));  7:         filteredPatternList.remove(P(*j*));  8:       **Else**
 9:        P(*j*).similarlist.add(P(*i*));  10:         filteredPatternList.remove(P(*i*));  11:       **EndIf**
 12:     **EndIf**
 13:   **EndFor**
 14: **EndFor**
 15: **Return** filteredPatternList

### 4.2. Generating Predictive Rules

Since the Apriori algorithm is a classic association rule mining algorithm which is simple, easy to understand and implement, and can obtain the correlation rules between conditional variables and decision variables, so the Apriori algorithm is used it to obtain correlation rules on these interesting patterns. The details of the Apriori algorithm are described in [38] and are not repeated here. Since the rule mining technique does not consider the semantics of the rules, in this paper we apply some filtering as a post-processing step to make sure the rules conform to domain constraints. In this step, based on the application requirements, we define a set of filters for the output rules. In the earthen ruin monitoring dataset, we have a set of patterns discovered in the air temperature data and another set of patterns from rammed earth temperature data. In this case, the rule miner finds all the relationships between patterns regardless of the conditional variable of the patterns. It is then possible to have some rules, such as $r=p_{air\_3}\to p_{air\_5}$, where the conditional and decision patterns are air temperature data. Since the target of the current study is finding relationships between patterns from air temperature and rammed earth temperature, rules, such as $r=p_{air\_3}\to p_{air\_5}$, are of no use. Therefore, we can define a filter to remove all the rules that have patterns from the same variable.

We then calculate the direction of each rule. We filter the rules whose directions are −1 or 0, because these rules are often due to the existence of some data anomalies or noise. We also calculate the delay and variation of each filtered rule and merge the above rules into more succinct predictive rules. The predictive rule may be in the following form:

If $\min(p_{air\_i})>0\;{}^{\circ}C\;$and $+5\;{}^{\circ}C\leq V(p_{air\_i})\leq +\;10\;{}^{\circ}C$
then $+2\;{}^{\circ}C\leq V(p_{earth\_j})\leq \;+3\;{}^{\circ}C$ with delay = 3 h

### 4.3. Predicting with Predictive Rules

After obtaining the prediction rule, we can use the time series of conditional variables to predict the time series of decision variables. The patterns are extracted in the time series of conditional variables to match the prediction rules based on the predictive rules. The matching results are used as the predictive results of the time series of decision variables. Hit rate H is defined to show the performance of the proposed algorithm:

(8) $$H=\frac{N}{M}$$

where numerator N is the number of the air temperature patterns that accurately predict the rammed earth temperature pattern, and denominator M. is the number of all the air temperature patterns in the testing dataset.

## 5. Experiments

**Figure 7:**
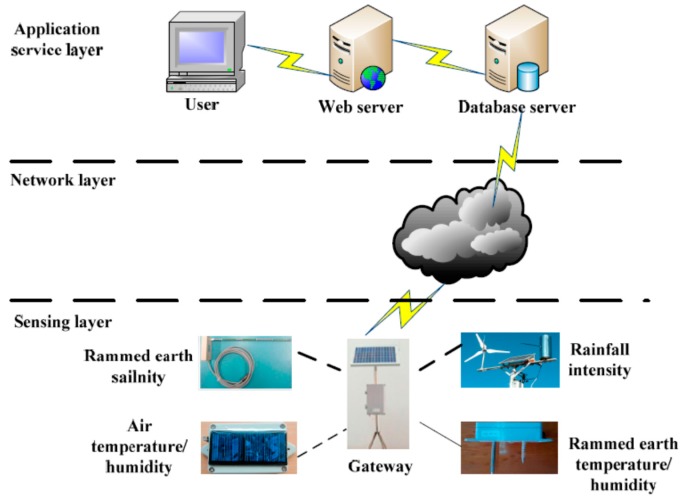
The IoT based sensing infrastructure for Ming Dynasty Great Wall.

To evaluate the proposed PPER method, we applied it to the Ming Dynasty Great Wall dataset monitored from the Ming Dynasty Great Wall in Shaanxi Province of China by an IoT network. We have deployed about 300 IoT nodes in five monitoring areas of the Ming Dynasty Great Wall in Shaanxi. The IoT based sensing infrastructure for the Ming Dynasty Great Wall is shown in Figure 7.

**Figure 8:**
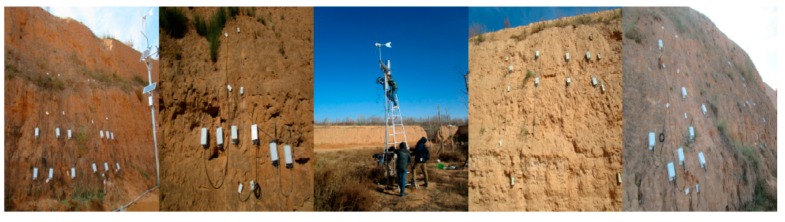
Photos of IoT nodes layouts in the 5 monitoring areas.

The sensing infrastructure includes three layers, sensing layer, network layer and the application service layer. In the sensing layer, there are different IoT nodes which can obtain environmental monitoring data, such as air temperature and humidity, and rainfall intensity, and earthen ruin monitoring data, such as rammed earth temperature, humidity and salinity. With Zigbee transmission technology, these nodes can transmit data to the gateway. In the network layer, with GPRS technology, data are finally reached the database server. A parser is developed to parse the received packets, and save the data into a database using MySQL Server. In the application service layer, user can visit the web server to query the data and view the analysis results. Some photos of the IoT node layouts in the five monitoring areas are shown in Figure 8.

In this work, we focused on obtaining rammed earth temperatures; therefore, we used the air temperature, rainfall intensity, rammed earth slope and aspect and rammed earth temperature monitoring data of the Ming Dynasty Great Wall dataset. The dataset consists of the air temperature, the rainfall intensity and the rammed earth temperature underground 5 cm in 5 monitoring areas collected from 29 October 2015 to 29 January 2016. It also consists of the rammed earth slope and aspect of each monitoring area. The air temperature monitoring frequency and the rainfall intensity were once every five minutes, and the rammed earth temperature monitoring frequency was once every three hours. The air temperature, the rainfall intensity and rammed earth temperature underground 5 cm of each monitoring area are considered as a multivariate sequence.

The algorithm was implemented in Java, and all experiments were run on a Windows 7 platform with a CPU speed of 3.4 GHz × 2, and 12 GB RAM. For the last part of the algorithm, which looks for potential relationships in the data, we used Weka library version 3.7.9.

### 5.1. Parameter Setting

The window size was set to 4 for the Ming Dynasty Great Wall dataset. The $\delta_{min}$ parameter was used to determine whether the algorithm discarded the sliding window or not. For air temperature data, $\delta_{min}$ was set to be 5, according to the minimum air temperature difference between day and night. For the rammed earth temperature data, $\delta_{min}$ was set to be 1, according to the minimum difference of the rammed earth temperature between day and night. For the rainfall intensity,$\;\delta_{min}$ was set to be 1, according to the minimum rainfall intensity difference between day and night. The overlapping length parameter was also set to 1, because the two adjacent interest patterns had a point overlap. The $\tilde{d}$ parameter was used to decide which patterns were similar. The smaller the $\tilde{d}$ parameter, the more the similar patterns, and vice versa. Therefore, $\tilde{d}$ was set at 18 for the air temperature sequence, 8 for the rammed earth temperature, and 0.1 for the rainfall intensity. Using the Ming Dynasty Great Wall dataset, we designed two sets of experiments to evaluate PPER.

### 5.2. Experiment on the Performance of Pruning Rules

**Figure 9:**
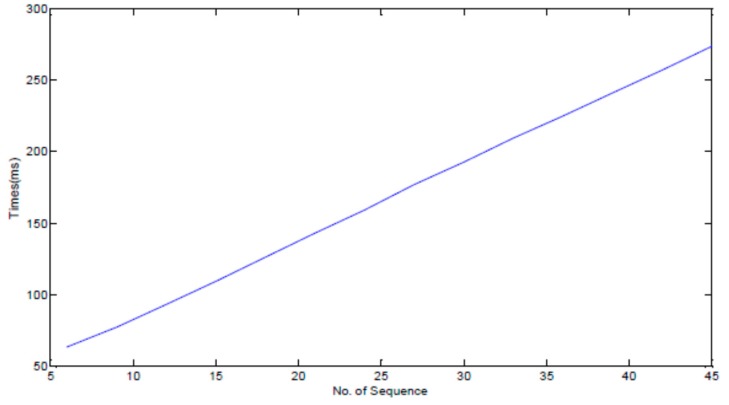
Performance of FindCandPatterns Algorithm**.**

**Figure 10:**
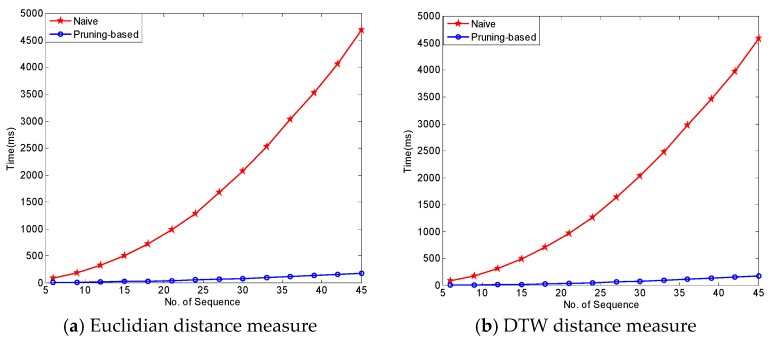
Performance of naïve and pruning-based distance matrix calculation algorithms vs. number of sequences.

To prove the effect of pruning rules in reducing the time complexity of the algorithm, we utilized air temperature in the Ming Dynasty Great Wall dataset. In this experiment, we evaluate the performance of FindCandPatterns method using Ming Dynasty Great Wall dataset. The performance of FindCandPatterns Algorithm is showed in Figure 9. As shown in this figure, the time complexity increases linearly with the number of sequences. The experimental results are consistent with the results of our time complexity analysis in Section 4.1.1. We then conducted some experiments to investigate the performance of the distance matrix calculation with and without pruning rules. As Figure 10 shows, the performance of the pruning-based algorithm is much better than the naïve one for both Euclidian and DTW distance measures.

**Figure 11:**
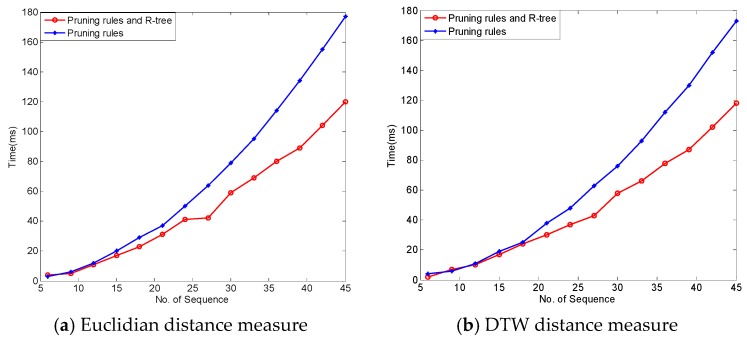
Effect of pruning levels on the performance of distance matrix calculation algorithms.

Figure 11 presents the results of another experiment—one which studied the effect of two levels of pruning on the performance of the distance matrix calculation algorithm. In the figure, the blue-colored line with ○ shows the algorithm that only applied index and slope pruning rules without using the R-tree-like data structure; and, the red line with * shows the results related to the version of the algorithm that utilizes the R-tree-like data structure for further pruning. It can be seen that for almost all situations, use of the R-tree improved the performance of the calculations. However, for distance matrix calculation, using the R-tree like structure does not lead to a huge performance improvement.

### 5.3. Experiments on the Performance of Prediction

In this work, the Apriori algorithm was used to discover correlation rules from the conditional variables patterns and the decision variable patterns in the Ming Dynasty Great Wall dataset. We selected the monitoring data of the partial nodes with the same orientation of south; half of these data were used to mine the correlation rules, and the other half were used to test the pattern prediction function of the algorithm.

In this experiment, we applied PPER in a dataset with three conditional variables, including air temperature rammed earth slope aspect and rainfall intensity, and obtained 42 the correlation rules. Among all discovered rules, the following example correlation rule describes the relationship among three conditional variables and one decision variable, i.e., the air temperature, rammed earth slope and aspect, and the rainfall intensity, and the rammed earth temperature:

(9) $${\;r}_{5}=p_{\mathrm{air}\_8}\;,{\;p}_{\mathrm{rain}\_9},p_{\mathrm{slope}\_1}\Rightarrow p_{\mathrm{earth}\_9},$$

where $p_{\mathrm{air}\_8}=\left(7.9,\;8.5,\;10.3,\;11.5,\;13.9\right)$, $p_{\mathrm{earth}\_9}=\left(2.1,\;2.3,\;2.7,\;3.5,\;4.3\right)$, $p_{\mathrm{slope}\_1}=\left(\;170\right)$ and $p_{\mathrm{rain}\_9}=\left(0.01,0.01,\;0,\;0,\;0\right)$. It shows when the air temperature increases from 7.9 to 13.9 °C, and the rainfall intensity decreases from 0.01 to 0, the rammed earth slope is toward the south with its aspect of 170 degrees, the temperature of rammed earth increases from 2.1 to 4.3 °C. Because precipitation in our study area is low, so the rainfall intensity data are sparse and does not have much variations, so the number of interesting patterns extracted based on the rainfall intensity are small, which does not provide too much in-depth knowledge to the earthen ruins conservation experts. Therefore, we continue analyzing the rules with air temperature as the conditional variable to predict the rammed earth temperature in order to solve the rammed earth temperature prediction with good performance. After keeping the correlation rules that describe the relationship from the air temperature to the rammed earth temperature and filtering the rules that had a negative direction, we determined 39 correlation rules with a confidence of 0.9.

**Figure 12:**
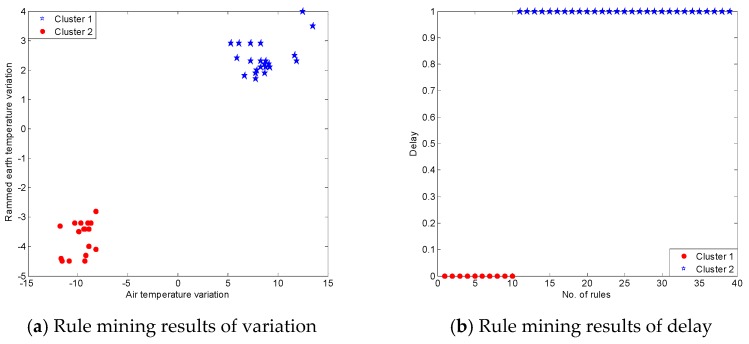
Rule mining results for Ming Dynasty Great Wall dataset.

K-means is a commonly used clustering method which is efficient and fast with the time complexity O(n), where n is the number of data objects. In this application, we use k-means method to analysis the variation and delay of these correlation rules. Because we focus on the two kinds of patterns, falling and rising patterns, so the number of cluster k is set as 2. We obtained the results shown in Figure 12. In Figure 12a, all the rising rules were placed into cluster 1 with air temperature variation from 5.3 °C to 13.5 °C and rammed earth temperature variation from 1.7 °C to 4 °C; and, all falling rules were placed into cluster 2 with air temperature variation from −11.8 °C to −8.2 °C and the rammed earth temperature variation from −4.5 °C to −3.2 °C. Figure 12b shows the two clusters of the delay of the 39 rules. About 74% of the delay was an observation point of time of about 3 h.

We continued to merge the above rules into more succinct predictive rules, as shown in Algorithm 4. There were six predictive rules that could be used to predict the rammed earth temperature pattern by rule matching.

**Algorithm 4.** Predictive Rules.**Predictive Rules**1: **If**
$\min(p_{air\_i})>0\;{}^{\circ}C\;$and $+5.3{}^{\circ}C\leq V(p_{air\_i})\leq +9.2\;{}^{\circ}C$

**then**
$+2.1\;{}^{\circ}C\leq V(p_{earth\_j})\leq \;+2.9\;{}^{\circ}C$ with delay = 3 h  2: **If**
$\min(p_{air\_i})>0\;{}^{\circ}C\;$and $-11.7{}^{\circ}C\leq V(p_{air\_i})\leq -8.9\;{}^{\circ}C$

**then**
$-\;4.5{}^{\circ}C\leq V(p_{earth\_j})\leq -4\;{}^{\circ}C$ with delay = 3 h  3: **If**
$\min\left(p_{air\_i}\right)\leq 0\;\;{}^{\circ}C\leq \max\left(p_{air\_i}\right)\;$and $+8.8\;{}^{\circ}C\leq V(p_{air\_i})\leq +11.9\;{}^{\circ}C$

**then**
$+2.1\;{}^{\circ}C\leq V(p_{earth\_j})\leq +2.3\;{}^{\circ}C$ with delay = 3 h  4: **If**
$\min\left(p_{air\_i}\right)\leq 0\;{}^{\circ}C\leq \max\left(p_{air\_i}\right)\;$and $-9.9\;{}^{\circ}C\leq V(p_{air\_i})\leq -8.7\;{}^{\circ}C$

**then**
$-3.5\;{}^{\circ}C\leq V(p_{earth\_j})\leq -3.2\;{}^{\circ}C$ with delay = 3 h  5: **If**
$\max\left(p_{air\_i}\right)\leq 0\;{}^{\circ}C\;$and $+5.9{}^{\circ}C\leq V(p_{air\_i})\leq +13.5\;{}^{\circ}C$

**then**
$+1.7\;{}^{\circ}C\leq V(p_{earth\_j})\leq +3.5\;{}^{\circ}C$ with delay = 3 h  6: **If**
$\max(p_{air\_i})\leq 0\;{}^{\circ}C\;$and $\;-11.8\;{}^{\circ}C\leq V(p_{air\_i})\leq -8.2\;{}^{\circ}C)$

**then**
$-3.3\;{}^{\circ}C\leq V(p_{earth\_j})\leq -2.8\;{}^{\circ}C$ with delay = 3 h

**Figure 13:**
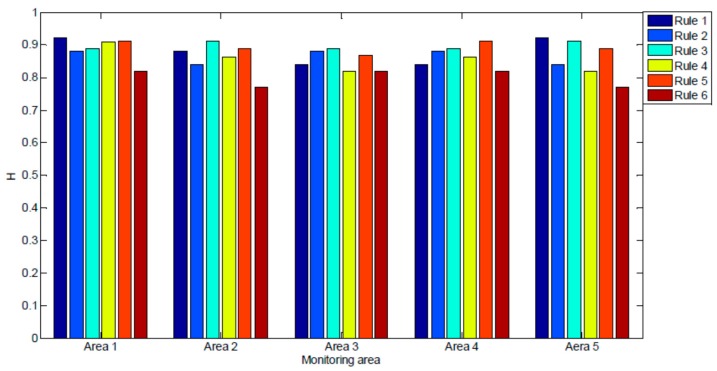
Prediction results for Ming Dynasty Great Wall dataset.

The hit rates of the rules are shown in Figure 13. We can observe that predictive rule 4 had the highest average hit rate (89.8%) and that predictive rule 6 had the lowest average hit rate (80.0%). In subsequent analysis, we found that predictive rule 3 merged 10 primitive correlation rules and predictive rule 6 only merged four primitive correlation rules; therefore, predictive rule 3 had the highest average hit rate.

## 6. Conclusions

In this paper, we investigated the problem of pattern prediction in data obtained from earthen ruins. We proposed the PPER pattern prediction method to find interesting patterns in air and rammed earth temperature sequences. In order to reduce the time complexity of the algorithm, we proposed two pruning rules and a new data structure. Using the correlation rules between the air and rammed earth temperature patterns, predictive rules were obtained. The rammed earth temperature patterns were well predicted with the predictive rules from the Ming Dynasty Great Wall dataset, which has great significance for the protection of the Ming Dynasty Great Wall. We have integrated PPER into the intelligent platform of Ming Dynasty Great Wall, and made a demonstration application in the Ming Dynasty Great Wall in Zhenbeitai, Shaanxi Province, China, and achieved good prediction results, which provided scientific decision-making for the protection of Ming dynasty Great Wall. PPER can be applied on multivariate problems. It can be extended to multiple pattern prediction with similar rising and falling interest patterns, such as the petroleum well log data, to predict the product rate pattern with pressure pattern. In the future, we will collect more monitoring data to improve the prediction performance of PPER. As the same time, we will extend and apply PPER to other multivariate problems. We plan to do more experiments and research to improve the efficiency and effectiveness of PPER. The generalizability of the algorithm will also be a focus of follow-up work, we plan to definite more interesting patterns to solve different pattern prediction.
